# Late Toxicities, Failure Patterns, Local Tumor Control, and Survival of Esophageal Squamous Cell Carcinoma Patients After Chemoradiotherapy With a Simultaneous Integrated Boost: A 5-Year Phase II Study

**DOI:** 10.3389/fonc.2021.738936

**Published:** 2021-11-18

**Authors:** Chuangzhen Chen, Jianzhou Chen, Ting Luo, Siyan Wang, Hong Guo, Chengbing Zeng, Yanxuan Wu, Weitong Liu, Ruihong Huang, Tiantian Zhai, Zhijian Chen, Derui Li

**Affiliations:** ^1^ Department of Radiation Oncology, Cancer Hospital of Shantou University Medical College, Shantou, China; ^2^ Oncological Research Lab, Cancer Hospital of Shantou University Medical College, Shantou, China; ^3^ Department of Radiation Oncology, Shenshan Central Hospital, Sun Yat-Sen Memorial Hospital, Sun Yat-Sen University, Shanwei, China; ^4^ Department of Radiation Oncology, Center for Cancer Prevention and Treatment, Meizhou People’s Hospital (Huangtang Hospital), Meizhou Academy of Medical Sciences, Meizhou Hospital Affiliated to Sun Yat-sen University, Meizhou, China; ^5^ Department of Radiation Oncology, National Cancer Center/National Clinical Research Center for Cancer, Cancer Hospital, Chinese Academy of Medical Sciences and Peking Union Medical College, Shenzhen, China

**Keywords:** esophageal cancer, chemoradiotherapy, simultaneous integrated boost, long-term outcomes, clinical trial

## Abstract

**Purpose:**

We aimed to evaluate the long-term outcomes of concurrent chemoradiotherapy (CCRT) with a simultaneous integrated boost (SIB) of radiotherapy for esophageal squamous cell carcinoma (ESCC).

**Methods and Materials:**

Eighty-seven patients with primary ESCC enrolled in this phase II trial. The majority (92.0%) had locoregionally advanced disease. They underwent definitive chemoradiotherapy. The radiotherapy doses were 66 Gy for the gross tumor and 54 Gy for the subclinical disease. Doses were simultaneously administered in 30 fractions over 6 weeks. The patients also underwent concurrent and adjuvant chemotherapy, which comprised cisplatin and fluorouracil. The study end points were acute and late toxicities, first site of failure, locoregional tumor control, and overall survival rates.

**Results:**

The median follow-up time was 65.7 (range, 2.2-97.5) months for all patients and 81.5 (range, 19.4-97.5) months for those alive. There were 17 cases (19.5%) of severe late toxicities, including four cases (4.6%) of grade 5 and seven (8.0%) of grade 3 esophageal ulceration, four (4.6%) of grade 3 esophageal stricture, and two (2.3%) of grade 3 radiation-induced pneumonia. Twenty-three (26.4%) patients had locoregional disease progression. Most (86.7%) locally progressive lesions were within the dose-escalation region in the initial radiation plan, while majority of the recurrent lymph nodes were found out-of-field (83.3%) and in the supraclavicular region (75.0%). The 1-, 2-, 3-, and 5-year locoregional tumor control and overall survival rates were 79.2%, 72.4%, 72.4%, 70.8%, and 82.8%, 66.6%, 61.9%, 58.4%, respectively. Incomplete tumor response, which was assessed immediately after CCRT was an independent risk predictor of disease progression and death in ESCC patients.

**Conclusions:**

CCRT with SIB was well tolerated in ESCC patients during treatment and long-term follow-up. Moreover, patients who underwent CCRT with SIB exhibited improved local tumor control and had better survival outcomes compared to historical data of those who had standard-dose radiotherapy.

## Introduction

Esophageal cancer (EC) is the sixth leading cause of cancer-related deaths worldwide (1). Concurrent chemoradiotherapy (CCRT) has been established as the standard treatment for locally advanced EC since the 1990s (2, 3). It consists of a total dose of 50.4 Gray (Gy) irradiation given in conventional fractionation to both the gross tumor and the subclinical disease with concurrently cisplatin-based chemotherapy. Despite CCRT, approximately 50% of patients still succumbed to locoregional tumor progression, with the majority of uncontrolled diseases found within the gross tumor volume (GTV) in the initial treatment plan (4). The INT0123 (Radiation Therapy Oncology Group (RTOG) 94-05) study examined radiation dose escalation in EC. In this study, an additional 14.4 Gy was administered to high-risk regions after the completion of standard-dose radiotherapy (RT) (50.4 Gy). However, patients who received a higher RT dose exhibited neither a greater overall survival (OS) nor tumor local control compared with those receiving standard-dose RT (5). Similarly, administration of a brachytherapy boost after external-beam radiotherapy (EBRT) did not improve survival in patients with EC. In addition, it was associated with an increased incidence of esophageal fistula (6, 7). Thus, the current non-surgical standard treatment for EC remains to be CCRT with a total radiation dose of 50.4 Gy in conventional fractionation. Moreover, local recurrence within the GTV remains to be the most common failure after treatment.

Modern RT, such as intensity modulated radiotherapy (IMRT) and image-guided radiotherapy (IGRT), can precisely and accurately deliver RT to target volumes, resulting in better organs at risk (OARs) sparing and target volume coverage. Additionally, it provides greater capability for radiation dose escalation in high tumor burden regions. One commonly used approach is simultaneous integrated boost (SIB), wherein a higher dose in moderated hypofractionation (> 2 Gy per fraction [F]) is delivered to the GTV, while a low dose in conventional fractionation is administered to the clinical target volume (CTV) (8, 9). SIB-based IMRT (SIB-IMRT) enables an increase in the overall RT dose administered to high-risk regions without prolonging the total treatment time. This approach has been examined in various cancer diseases, including head and neck cancer, anal cancer, and prostate cancer, and has been shown to improve tumor local control (10–20).

Due to the emerging clinical value of SIB-IMRT in the treatment of cancer, there has been a growing interest in revisiting RT dose escalation in EC. Previous studies conducted in the 1990s that examined RT dose escalation in EC mainly used two-dimensional and sequential radiation delivery technique which had its disadvantages such as prolonged treatment time, poor sparing of OARs from high-dose irradiation, and uncertainty of tumor coverage. In contrast, SIB-IMRT can accurately and precisely deliver augmented RT dose to well-defined GTVs in a relatively shorter period of time, which may help eliminate radiation-resistant cancer cell clones, and to some extent prevent accelerated tumor clonogen repopulation (21, 22). Its greater tissue sparing effect could help avoid the significantly increased risk and severity of radiation-induced toxicities (23–25). Collectively, these advantages could help expand the therapeutic window for RT in patients with EC, thus resulting in better outcomes. The feasibility of SIB-IMRT in EC was first reported by radiation dosimetric studies followed by its examination in a number of phase I/II clinical trials (24–28). These studies suggested that SIB-IMRT combined with chemotherapy was feasible in EC patients with tolerable short-term toxicities and showed an improving trend of local tumor control.

Despite these promising results in earlier studies that investigated the short-term effects of SIB-IMRT, its long-term effects on efficacy and tolerability remain unclear. In SIB-IMRT, esophageal tissues within or close to the gross tumor are irradiated with a much higher dose compared with their counterparts in standard-dose RT. Its late toxicities such as esophageal ulceration and stricture can significantly affect the quality of life of long-term survivors. In the INT0123 study, a higher radiation dose was associated with severe toxicities, although no causal correlation was established (5). Nevertheless, the moderate hypofractionation utilized by SIB together with concomitant chemotherapy render the esophagus a critical OAR and made patient tolerability uncertain. In addition, it was still unknown whether improvement of local tumor control at an early stage could translate into a long-term survival benefit. Furthermore, target volume delineation with various extent of shrinkage was utilized in these studies. Its utilization required validation on the basis of disease control rates as well as tumor failure patterns. Addressing these questions could provide a comprehensive evaluation of the value of SIB-IMRT in EC. In this study, we conducted a phase II clinical trial to examine definitive CCRT with radiation SIB in patients with esophageal squamous cell carcinoma (ESCC). The endpoints we examined were acute and late toxicities, failure patterns, 5-year locoregional control (LRC), and OS rates.

## Methods and Materials

### Patient Selection and Evaluation

The inclusion criteria were as follows: (a) histologically or cytologically confirmed primary ESCC; (b) lesion located in the cervical, upper thoracic, or middle thoracic esophagus; (c) absence of distant metastasis (except for supraclavicular (SCV) lymph node (LN) metastasis) and other malignant tumors; (d) Zubrod performance status score of 0 to 2; (e) adequate liver, renal, and bone marrow functions; and (f) women of childbearing potential and men practicing contraception.

The exclusion criteria were as follows: (a) tracheoesophageal or mediastinal esophageal fistula; (b) history of invasive malignancy (except for non-melanomatous skin cancer) unless disease-free for a minimum of 2 years; (c) overlapping of previous RT fields with the currently planned ones; (d) severe comorbidities; and (e) pregnant or nursing women. This study was approved by the local Clinical Ethnic Committee at 201833 and registered at Clinicaltrial.gov (NCT01670409). Informed consent was obtained from all enrolled patients.

The following evaluations were performed prior to treatment: (a) history and physical examination; (b) complete blood count; (c) blood biochemistry; (d) esophageal endoscopic ultrasonography (EUS) with biopsy; and (e) imaging examinations including barium esophagram, plain and contrast-enhanced computed tomography (CT) of the neck, chest, and abdomen, and abdominal ultrasound. Bronchial endoscopies, bone scans and positron emission tomography-CT (PET/CT) scans were performed as clinically indicated. All patients were staged according to the American Joint Cancer Committee (AJCC) staging system 6^th^ (29).

### RT

RT was delivered using the SIB-IMRT approach. Target volume delineation was based on available imaging resources such as contrast-enhanced CT scans, endoscopic reports, barium esophagram, and PET/CT. GTVs included both the primary esophagus tumor (GTV_P_) and positive LNs (GTV_LNs_). Mediastinal and supraclavicular LNs were considered positive if their shortest axis is greater than or equal to 1 cm. The CTV was contoured by adding a 2.0 cm longitudinal and 0.5 to 1.0 cm radial margins to the GTV_P_ and a 0.5 cm uniform margin to the GTV_LNs_. Esophagotracheal LNs were included in the CTV if their shortest axis was between 0.5 and 1.0 cm. The PTV66 and the PTV54 were generated from the GTVs and the CTV with a 0.5 cm expansion margin, respectively. The prescribed doses were 66 Gy (2.2 Gy/F) to the PTV66 and 54 Gy (1.8 Gy/F) to the PTV54, which were simultaneously delivered in 30 F (1 F per day). OARs included the spinal cord, heart, and lungs.

Treatment plans were designed by using the sliding-window IMRT with five coplanar 6-MV photo beams (angles: 210°/300°/0°/60°/150°). The planning objectives for the PTV were 95% of its volume receiving the prescribed dose, with V_107_ < 5% and V_93_ < 1% (V_n_ = percentage of the PTV covered by n% of the prescribed dose). The dose constraints of OARs were as follows: spinal cord, D_max_ (maximum dose) < 45 Gy; heart, V_40_ (V_x_= percentage of the target volume receiving ≥ x Gy) < 100%, V_45_ < 67% and V_50_ < 33%; lungs, V_20_ < 30%, V_10_ < 50%, and V_5_ < 60%. Dose calculation was performed using the Anisotropic Analytical Algorithm 8.6.15 with lung heterogeneity correction.

RT was delivered by a linear accelerator (TrueBeam, Varian Medical Systems, Palo Alto, CA) with a maximum dose rate of 600 MU/min. All patients underwent 3D cone-beam CT imaging (CBCT) at least once per week prior to treatment for evaluation and correction of potential setup errors.

### Chemotherapy

Patients received two cycles of concurrent chemotherapy on the first and fifth weeks, and another two cycles of adjuvant chemotherapy on the eighth and eleventh weeks. The chemotherapy regimen consisted of intravenous cisplatin (75 mg/m^2^) on the first day and intravenous fluorouracil (0.5 g/m^2^) from the first to the fourth day.

### Toxicity Evaluation and Management

Therapeutic-related toxicities were scored using the Common Terminology Criteria for Adverse Events (CTCAE, version 4.0) (30). Toxicities were managed with supportive care and dose modifications. Dose modifications consisted of dose reduction, treatment delays, and treatment cancellation depending on the severity of toxicity.

### Follow-Up

Patients were evaluated weekly during CCRT. After treatment, they were followed up every three months over a period of two years, every six months within the next three years, and every year thereafter. Follow-up evaluations consisted of history, physical examination, blood test, chest X-ray or contrast-enhanced CT scan, esophagography, and abdominal ultrasound. Esophageal EUS, PET/CT, or electrocardiogram was performed if they were indicated. Local recurrence was confirmed by pathological examination or by at least two imaging examinations when pathological proof was not available. Longitudinal imaging was used to evaluate regional recurrence and distant metastasis excluding other malignant diseases.

### Endpoints

The primary endpoint of this phase II study was patient tolerability of CCRT with radiation SIB in patients with ESSC. Its evaluation consisted of the completion of treatment and acute and late therapeutic-induced toxicities in patients. Secondary endpoints included the first site of failure; 1-, 2-, 3- and 5-year locoregional control (LRC); distant metastasis-free survival (DMFS); disease progression-free survival (DPFS); and overall survival (OS). Tumor response was assessed by using the Response Evaluation Criteria in Solid Tumors, version 1.1 (RECIST1.1) (31). Failure patterns at locoregional sites were determined by matching follow-up and baseline examinations, including CT scan, barium esophagram, and endoscopic ultrasonography-guided biopsy. Recurrent lesions that were either entirely or partially within the target volume in the initial treatment plan were defined as in-field progression, whereas the remaining was considered as out-of-field progression.

### Statistical Methods

The sample size calculation for this study has been described in previous report (32). Overall, 85 patients had an 80% of power to detect a significant (α < 0.05) increase of 2-year LRC by 15% from 50% in historical data. Time-to-event was measured from the date when RT was started to the date of event occurrence and last clinic visit. The events were locoregional disease progression, distant metastasis, and death. The survival curves were generated using the Kaplan–Meier method. Statistical calculations were performed using the Statistical Package for Social Sciences software, version 23.0 (Chicago, IL). The comparison between the RT dose in our study and those of others was performed using the biologically effective dose (BED), which was calculated using the following formula: BED = Nd x (1 + d/(α/β)), N for the number of fractions, d for RT dose per fraction, α/β = 10 (33). Univariate analysis for the 5-year cumulative risk of disease progression or death in subgroups of patients was performed using the log-rank’s test. Patients were categorized according to their clinical characteristics (e.g., age, sex, tumor location, stage, and tumor volumes), treatment (e.g. chemotherapy cycles), and short-term response (response-20 F: as assessed immediately after 20 F; response-30 F: assessed after 30 F). Parameters selected from univariable analysis were included in the multivariable cox regression (with the enter selection method) analysis for identifying independent predictors. A *P* < 0.05 was considered statistically significant.

## Results

### Patient Characteristics

Between August 2012 and August 2015, 87 ESCC patients were enrolled in this single-arm phase II trial, which was the most current as of writing on the 30^th^ of August, 2020. The median follow-up time was 65.7 months (range, 2.2-97.5 months) for all patients and 81.5 months (range, 19.4-97.5 months) for those still alive. The 5-year follow-up rate was 97.7%. Only two patients (2.3%) were lost to follow-up 19.4 and 32.1 months after their start of treatment. All patients enrolled in this trial were included in this analysis. Patient clinical characteristics are summarized in **Table 1**. Approximately 92.0% of patients had locoregionally advanced diseases (T_3-4_ or N+), while 19.5% had positive SCV LNs (M_1_).

**Table 1:** Clinical characteristics of 87 esophageal cancer patients underwent definitive chemoradiotherapy with radiation simultaneous integrated boost.

Characteristics		No.	%
Age (years)	≤ 60	42	48.3
	> 60	45	51.7
Gender	Male	67	77.0
	Female	20	23.0
T stage*	T1	1	1.1
	T2	17	19.5
	T3	40	46.0
	T4	29	33.3
N stage*	N0	31	35.6
	N1	56	64.4
M stage*	M0	74	85.1
	M1	13	14.9
Clinical stage*	II	29	33.3
	III	45	51.7
	IV	13	14.9
Lesion site	Cervical	7	8.0
	Upper thoracic	35	40.2
	Middle thoracic	45	51.7
ECOG Score	0	1	1.1
	1	79	90.8
	2	7	8.1

ECOG, Eastern Cooperative Oncology Group.

*According to the American Joint Cancer Committee (AJCC) staging system 6^th^.

### Treatment Completion

The treatment planning targets were met in all enrolled patients. Almost all patients (98.3%) completed the entire course of SIB-IMRT. Only one patient (1.7%) missed a fraction (3% of the prescribed dose) of RT due to grade 4 thrombocytopenia. Most patients (95.4%) completed two cycles of concurrent chemotherapy and about 85.1% of patients received at least one cycle of adjuvant chemotherapy. A total of 23 patients (26.4%) had chemotherapy cancellation due to the following reasons: patient’s refusal in eight patients (34.8%); severe bone marrow depression in eight (34.8%); esophageal ulceration/fistula in two (8.7%); esophagitis in two (8.7%); vomiting in two (8.7%); and distant metastasis in one (1.1%). A total of two patients (2.3%) refused adjuvant chemotherapy and underwent exploratory thoracotomy and gastrostomy based on their own decisions. Both were included in the intent-to-treatment analysis. There was no dose modification for chemotherapy.

### Toxicities

Acute and late toxicities experienced by patients are summarized in **Table 2**. In total, 24 patients (28%) displayed grade 3-4 acute toxicities during treatment, while 17 (19.5%) experienced severe (≥ grade 3) late toxicities within the 5-year follow-up. All grade 5 esophageal ulceration/fistula occurred in the first year of follow-up. Patients with grade 3 late toxicities responded well to clinical intervention and supportive care. Matching of follow-up CT scans to those used for the initial treatment planning suggested that esophageal ulcerations were within the PTV66. Further dosimetry analysis did not find apparent differences in RT dose distribution at the dose escalation region between patients with ulcerations and those without. In particular, the V_110_ (> 72.6 Gy) in PTV66 was less than 2% in all cases. No severe late toxicities of the heart, spinal cord, skin, and liver or other organs were reported in this study.

**Table 2:** Acute and late toxicities of 87 esophageal cancer patients after definitive chemoradiotherapy with radiation simultaneous integrated boost.

Toxicities No (%)	Grade 3	Grade 4	Grade 5
Acute			
Neutropenia	7 (8.0)	5 (5.7)	0
Esophagitis	4 (4.6)	0	0
Nausea/Vomiting	4 (4.6)	0	0
Thrombopenia	2 (2.3)	2 (2.3)	0
Others	0	0	0
Late			
Esophageal ulceration	7 (8.0)	0	4 (4.6)
Esophageal stricture	4 (4.6)	0	0
Lung	2 (2.3)	0	0
Heart	0	0	0
Spinal cord	0	0	0
Others	0	0	0

Toxicities were scored by using the Common Terminology Criteria for Adverse Events (CTCAE, version 4.0)

### First Site of Failure and Tumor Control Rates

**Figure 1:**
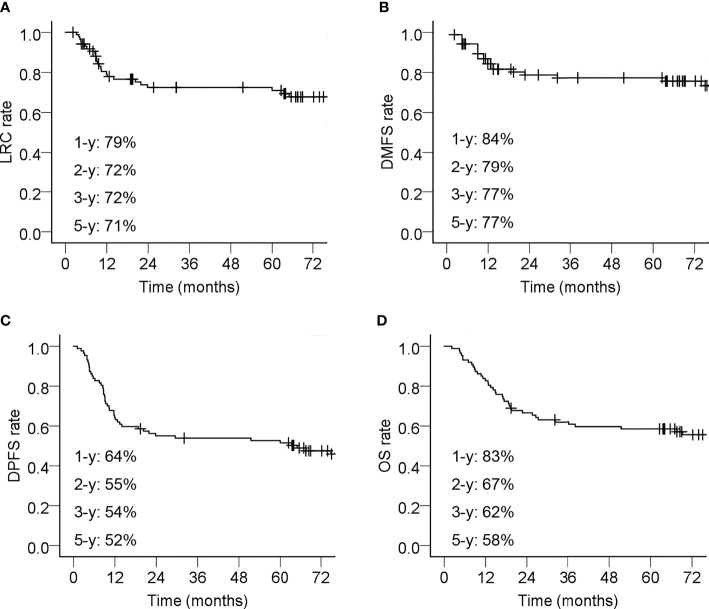
Kaplan–Meier curves of 87 esophageal cancer patients underwent definitive chemoradiotherapy with radiation simultaneous integrated boost. **(A)** Locoregional control (LRC) rate; **(B)** Distant metastasis-free survival (DMFS) rate; **(C)** Disease progression free survival (DPFS) rate; **(D)** Overall survival (OS) rate. The 1-year (-y), 2-y, 3-y, and 5-y tumor control rates or survival rates were shown. Censored events are indicated with ‘‘+’’ in the curves.

The short-term responses of tumor after treatment (Response-30 F) in this cohort of patients were as follows: complete response (CR) in 35 cases (40.2%), partial response (PR) in 50 cases (57.5%), and stable disease (SD) in two cases (2.3). At the time of analysis, 35 patients (40.2%) had disease progression within 5 years after their treatment. Their first site of failure and the time of occurrence are summarized in **Table 3**. The incidences of failures at various sites of all patients were as follows: distant metastasis in 18 cases (20.7%), local in 15 cases (17.2%), and regional in 12 cases (13.8%). The clinical characteristics of patients with local or regional disease progression are summarized in **Tables S2** and **S3**. The 5-year LRC, DMFS, and DPFS rates were 71%, 77% and 55%, respectively (**Figures 1A–C**).

**Table 3:** First site of failure of 87 esophageal cancer patients after definitive chemoradiotherapy with radiation simultaneous integrated boost.

First site of failure	N	%	Occurrent time Median (range) month
Distant	13	14.9	8.7 (1.1-29.5)
Local	11	12.6	9.1 (4.2-60.0)
Regional	6	6.9	13.9 (3.2-14)
Local and regional	3	3.4	9.3 (4.6-11.9)
Regional and distant	2	2.3	8.0 (5.7-10.4)
Local-regional and distant	1	1.1	3.9

### Failure Patterns

**Figure 2:**
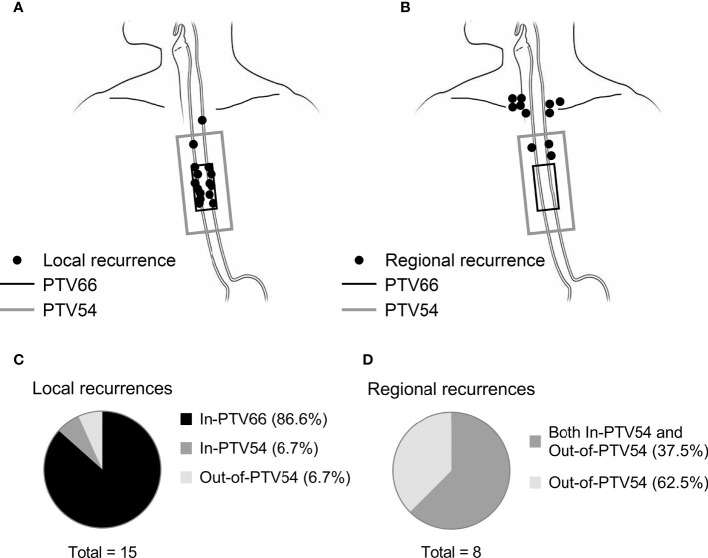
The location of local or solely regional recurrence in relation to the PTVs in esophageal cancer patients underwent definitive chemoradiotherapy with radiation simultaneous integrated boost. The location of local (n = 15) and solely regional (n = 8) recurrence in relation to PTV66 (in black) and PTV54 (in gray) was shown in **(A, B)**, respectively. Each black point represents one case of relapse. The distribution of failure patterns was summary in **(C, D)**.

The distribution of local progressive lesions in relation to the PTVs was demonstrated in **Figures 2A, C**. Most (13/15; 87%) of them were In-PTV66 in the initial radiation plan. The overall risk of Out-of-PTV54 progression at the esophagus was 1.1% (1/87). A total of eight out of the 12 cases of regional recurrence occurred without concomitant local progression. Their locations are shown in **Figure 2B**. They were all Out-of-PTV54 with or without concurrently In-PTV54 relapse (**Figure 2D**). The overall risk of Out-of-PTV54 regional recurrence in this cohort of patients was 9.2%. All Out-of-PTV54 regional recurrences were found in the supraclavicular (SCV) region. The risks of SCV LN recurrence for patients with different primary tumor location were as follows: cervical, 28% (2/7); upper thoracic, 8.6% (3/35); and middle thoracic, 6.7% (3/45). Distant metastasis occurred mostly in the following sites: lungs (50.0%), bones (38.9%), liver (27.8%), and nonregional lymph nodes (27.8%).

### Cause of Death and OS

A total of 40 patients (40/87; 46.0%) died during the follow-up period. The causes of death included local recurrence (14/40, 35.0%), distant metastasis (10/40, 25.0%), esophageal hemorrhage (4/40, 10.0%), regional recurrence (3/40, 7.5%), non-esophageal cancer diseases (5/40, 12.5%), and unknown causes (4/40; 10.0%). The 5-year OS rate was 58% (**Figure 1D**).

### Predictors for Risk of Disease Progression or Death

The multivariable cox regression analysis results suggested that the Response-30 F was an independent predictor for the 5-year cumulative risk of disease progression or death in this cohort of patients (**Tables S4–11**). As summarized in **Table 4**, patients without CR (i.e., PR or SD) were associated with a significantly higher risk for locoregional disease progression, distant metastasis, disease progression, or even death as compared to patients with CR. A male sex was also an independent risk factor for disease progression (**Table S9**).

**Table 4:** Comparison of 5-year cumulative risk of disease progression or death between esophageal cancer patients with various response immediately after concurrent chemoradiotherapy with simultaneous integrated boost.

Type of risk	5-year cumulative risk (%)		RR (95% CI)	P
	CR (n = 35)	PR/ST (n = 52)		
LRPD	15	48	3.3 (1.2-9.2)	0.023
Distant metastasis	9	39	4.5 (1.3-15.7)	0.017
Disease progression	24	66	3.7 (1.7-8.3)	0.001
Death	24	64	2.9 (1.2-7.1)	0.020

LRPD, locoregional disease progression; CR, complete response; PR, partial response; ST, stable; RR, relative risk; CI, confidence interval.

## Discussion

Locoregional tumor control in EC undergone definitive RT remains an unmet clinical need. The predominant in-field failure pattern after a standard-dose RT, the technical advances in RT delivery, and the promising clinical outcomes of SIB in other types of cancer have prompted the revisiting of radiation dose escalation in EC. As shown in **Table 5**, the results of SIB-RT as reported from several phase II studies were compared with that of the INT0123 study (5, 26, 36). In this study, our long-term results suggested that patients who received this regimen exhibited tolerable late toxicities, promising locoregional tumor control, and improved survival compared to those in historical data.

**Table 5:** Summary of phase II/III clinical trials examining radiation dose escalation with external beam radiotherapy in esophageal cancer.

	INT0123 Control arm	INT0123 High-dose arm	Li et al. (34)	Chen et al. (35)	Our study
Patient No	109	109	44	46	87
SCC: AC	84%: 16%	87%: 13%	100%: 0%	52%: 48%	100%: 0%
TNM stage					
T3-4	43%	48%	81%	87%	79.3%
N1	17%	27%	98%	68%	64.4%
M1	-	-	-	17%	14.9%
Stage I-II			13%	22%	33%
Stage III-IV			87%	78%	67%
RT dose					
PTV-G	50.4Gy/28F 1.8Gy/F	64.8Gy/36F 1.8Gy/F	59.92Gy/28F 2.14Gy/F	63Gy/28F 2.25Gy/F	66Gy/30F 2.2Gy/F
PTV-C	50.4Gy/28F 1.8Gy/F	64.8Gy/36F 1.8Gy/F	50.4Gy/28F 1.8Gy/F	50.4Gy/28F 1.8Gy/F	54Gy/30F 1.8Gy/F
PTV-G BED2	61.74 Gy	79.4 Gy	76.0 Gy	80.7 Gy	84.2 Gy
Treatment time (weeks)	5.6	7.2	5.6	5.6	6
RT techniques	2D	2D	IMRT	IMRT	IMRT
Boost methods	NA	Sequential	SIB	SIB	SIB
Acute toxicities (Grade 3-5)	G3: 43% G4: 26% G5: 2%	G3: 46% G4: 21% G5: 9%	G3: 53% G4: 4% G5: 0	G3: 22% G4: 0 G5: 0	G3: 20% G4: 8% G5: 0
Late toxicities (Grade 3-5)	G3: 24% G4: 13% G5: 0	G3: 34% G4: 11% G5: 1%	G3: 0 G4: 0 G5: 2%	G3: 7% G4: 0 G5: 0	G3: 15% G4: 0 G5: 5%
OS rates	2-y: 40%	2-y: 31%	1-y: 76.9%	1-y: 78% 2-y: 41% 3-y: 29%	1-y: 83% 2-y: 67% 3-y: 62% 5-y: 58%
LC rates	2-y: 48%	2-y: 44%	1-y: 78.8%	1-y: 70% 2-y: 67%	1-y: 87% 2-y: 82% 3-y: 82% 5-y: 80%
Reference	5	5	26	28	

SCC, squamous-cell carcinoma; AC, adenocarcinoma; PTV-G, planning target volume for gross tumor; PTV-C, planning target volume for subclinical diseases; BED2, biologically equivalent dose for 2Gy conventional fractionated radiotherapy; 2D, two dimensional; IMRT, Intensity modulated radiotherapy; SIB, Simultaneous Integrated Boost; NA, not applicable; G, Grade; OS, Overall survival; LC, Local control; -y, -year.

A major concern for radiation dose escalation in EC is the toxicities of OARs, including the esophagus itself. In this study, most patients exhibited minor to moderate and manageable side effects of the treatment. Severe late toxicities of the esophagus were only found in 19.5% of patients. Of them, four cases (4.6%) had grade 5 esophageal ulceration, which was not apparently higher than those in previous studies (5, 26). Severe late esophagitis was not associated with a significantly higher irradiation dose in the esophagus compared to patients without. Methodologies are needed to identify high-risk patients in advance to allow early intervention immediately. Only four (4.6%) patients experienced grade 3 esophageal strictures in this cohort within five years after the start of RT. This indicated that SIB-IMRT did not lead to substantially increased risk of severe esophageal stricture as compared to that in patients receiving conventional RT in historic data (37–39).Consistent with our data, Chen et al. showed that SIB-RT was well tolerated in locally advanced EC (28). In a multicenter retrospective data analysis of 2132 patients reported by Wang et al., the results showed the incidence of late toxicity in SIB group were significantly lower than No-SIB group (40).These results suggested that SIB-IMRT in combination with concurrent chemotherapy was tolerable in EC patients.

The long-term results of this study proved the hypothesis that RT dose escalation using the SIB approach could help improve LRC in EC patients. The 2-, 3-, and 5-year LRC rates were 72.4%, 72.4% and 70.8%, respectively, which were higher than those in historic reports. In line with our results, Chen et al. demonstrated that SIB-RT was associated with promising tumor control (2-year local control (LC) rate: 67%) in a group of patients with mixed pathological types (28). Notably, negative results also emerged. Hulshof et al. conducted a phase III trial to examine CCRT with or without SIB (61.6 Gy/28 F, 2.2 Gy/F *vs* 50.4 Gy/28 F, 1.8 Gy/F) for the definitive treatment of EC (41). Patients who underwent SIB-RT showed a trend of improving LRC compared with those who received standard-dose RT, but the difference was not statistically significant (63% *vs* 53%, *P* = 0.08). SIB-RT also led to a substantial increase of toxicities. In comparing their results with ours, factors such as patient characteristics (e.g., disease stage and pathological type), radiation dose, and the incidence of severe toxicities should be considered. Patients enrolled in this study displayed a greater response and less severe toxicities compared with those in Hulshof et al.’s study despite having more patients with advanced stages (e.g., T_4_, N_1_ or M_1_). This discrepant observation might be attributed to different designs of radiation regimen.

The augmentation of locoregional tumor control was associated with improvement of OS in this study. The majority of locoregional tumor progression occurred within 12 months after treatment. The LRC and OS curves plateaued on the second and third years, reaching 72% and 62%, respectively. These promising results suggested that improvement in the locoregional tumor control through radiation dose escalation could successfully be translated into long-term survival benefits.

The patterns of locoregional failure in this study provided useful insights for future clinical studies. There were 15 cases (17.2%) of tumor relapses at local sites. The cumulative risk of out-of-PTV54 relapse at the esophagus was only 1.1%, albeit a relatively smaller CTV, thereby suggesting that CTV delineation was sufficient to encompass almost all subclinical diseases at the esophagus. Notably, most locally progressive lesions were found within the PTV66, while local disease progression was the main death reason of this cohort of patients. The predominant in-field failure pattern suggested that radiation resistance was the leading cause of tumor control failure and raised a question regarding whether further escalating the RT intensity could improve OS. The extent of escalation that would be required to eliminate radiation-resistant tumors is unknown. While additional RT dose is likely to increase the risk of severe esophageal toxicities in patients. Thus, further RT escalation might not be an appropriate option in patients with radiation-resistant tumors. Management of these patients required early identification and a combination of CCRT with other therapeutics, such as salvage surgery and immunotherapy. Short-term tumor response emerged as an independent predictor for locoregional disease progression. Therefore, it could help select EC patients for personalized adjuvant treatment after CCRT. Immunotherapy using immune checkpoint blockers could release the break of existing anti-tumor immune response, thus leading to the elimination of cancer cells in a systemic fashion (42). Adding ICBs to SIB-based CCRT might help further improve tumor control and OS in EC.

The overall incidence of solely regional recurrence was about 9.2% in this patient cohort, indicating that the radiation regimen and CTV contour for the LN draining region were sufficient to eliminate the majority of metastatic lymphatic diseases. Most Out-of-PTV54 regional recurrence was found in the SCV region. Thus, prophylactic SCV irradiation could be considered in subgroups of patients, considering their primary tumor location. Cervical EC patients were associated with a higher risk (28%) of SCV regional recurrence. Therefore, they needed to be routinely subjected to SCV prophylactic irradiation. Since the CTV in their treatment plan already encompassed a part of the SCV region, expanding the CTV to include the entire SCV region would not result in significant increase of irradiation to OARs. In contrast, patients with middle or lower thoracic EC were less likely to benefit from prophylactic SCV irradiation. Including SCV in the CTV might reduce the risk of regional recurrence in a small fraction of patients, but the majority of patients would be subjected to unnecessary irradiation with possibly a significant increase of toxicities.

## Conclusions

CCRT with SIB was tolerated by patients with EC over the period of long-term follow-ups. Patients exhibited an excellent 5-year LRC and OS compared to those in historical data who had standard-dose RT. Most locally progressive lesions were found within the PTV66, suggesting that novel strategies were warranted to tackle these radiation-resistant tumors. Patients with cervical EC might benefit from routinely prophylactic irradiation to the SCV region as they were more likely to suffer from SCV LN recurrence. Short-term tumor response (immediately after CCRT) was an independent predictor for disease progression or death in this cohort of patients and might help select patients for personalized adjuvant treatment after CCRT.

## Data Availability Statement

The raw data supporting the conclusions of this article will be made available by the authors, without undue reservation.

## Ethics Statement

The studies involving human participants were reviewed and approved by Clinical Ethnic Committee of Cancer Hospital of Shantou University Medical College. The patients/participants provided their written informed consent to participate in this study.

## Author Contributions

CC contributed to resources, conceptualization, data curation, software, formal analysis, supervision, funding acquisition, validation, investigation, visualization, methodology, writing–original draft, project administration, writing–review and editing. JC contributed to conceptualization, data curation, software, formal analysis, supervision, validation, investigation, visualization, methodology, writing–original draft, project administration, writing–review and editing. TL contributed to data curation, software, formal analysis, validation, investigation, visualization, methodology, writing–original draft. SW contributed to methodology, writing–original draft,writing–review and editing. HG, RH, and T-TZ contributed to resources and investigation. CZ, YW, and WL contributed to resources, validation and investigation. ZC and DL contributed to investigation and project administration. All authors contributed to the article and approved the submitted version.

## Funding

This study was funded by Shantou University Medical College Clinical Research Enhancement Initiative, N0201424 (to CC and JC); Science and Technology Special Fund of Guangdong Province of China, 2019-132 (to CC); Strategic and Special Fund for Science and Technology Innovatoin of Guangdong Province of China, 180918114960704 (to CC).

## Conflict of Interest

The authors declare that the research was conducted in the absence of any commercial or financial relationships that could be construed as a potential conflict of interest.

## Publisher’s Note

All claims expressed in this article are solely those of the authors and do not necessarily represent those of their affiliated organizations, or those of the publisher, the editors and the reviewers. Any product that may be evaluated in this article, or claim that may be made by its manufacturer, is not guaranteed or endorsed by the publisher.
