# Supplementary material for: Late Toxicities, Failure Patterns, Local Tumor Control, and Survival of Esophageal Squamous Cell Carcinoma Patients After Chemoradiotherapy With a Simultaneous Integrated Boost: A 5-Year Phase II Study
Source: Front Oncol. 2021 Nov 18;11:738936. doi: 10.3389/fonc.2021.738936 (PMC8639085; doi:10.3389/fonc.2021.738936)
Supplement: Supplementary file 1 [file DataSheet_1.pdf]

Median (range)	PTV66	PTV54
Volume (cm3)	77.4 (13.5-212.0)	204.2 (97.5-750.7)
D_2 (Gy)	69.2 (67.5-71.5)	67.9 (59.6-70.6)
D_{98} (Gy)	65.3 (62.9-66.1)	53.4 (50.4-55.7)
D_{50} (Gy)	67.9 (66.8-69.90)	60.2 (56.1-66.9)
HI	0.06 (0.03-0.11)	0.25 (0.11-0.30)
CI	0.80 (0.51-0.96)	0.83 (0.49-0.94)

Abbreviations: PTV: planning target volume. *HI*: Homogeneity Index. *CI*: Conformity index. V_T: The target volume. $V_{T,ref}$: The target volume covered by reference isodose. V_{ref}: The total volume covered by reference isodose.

Dx was defined as the minimum dose to a specified target volume. *HI* = D_5/D_{95}. D_5 and D_{95} were the minimum doses received by the hot 5% and cold 95% of PTV, respectively. *CI*: Conformity index =$(V_{T,ref}/V_T)\times(V_{T,ref}/V_{T,ref})$

Table S1. Target volumes and dosimetric data of intensity modulated radiotherapy with simultaneous integrated boost for 87 esophageal cancer patients.

No	Sex	Age	Tumor location	TNM stage *	Patterns	time (months)
1	male	50	middle	T2N1M1b	In PTV66	12
2	male	67	upper	T3N0M0	In PTV66	22
3	male	56	upper	T4N1M0	In PTV66	5
4	male	70	middle	T2N0M0	In PTV54	24
5	male	70	middle	T2N0M0	In PTV66	10
6	male	48	middle	T3N1M0	Out of PTV54	20
7	male	61	middle	T4N1M1b	In PTV66	9
8	male	57	middle	T4N1M0	In PTV66	4
9	male	52	middle	T3N1M0	In PTV66	9
10	female	50	middle	T4N1M0	In PTV66	4
11	male	47	middle	T4N1M0	In PTV66	9
12	male	72	middle	T3N1M0	In PTV66	5
13	female	53	upper	T2N0M0	In PTV66	60
14	male	54	upper	T3N1M0	In PTV66	6
15	male	64	upper	T3N1M0	In PTV66	9

Abbreviations: CR: complete response; PR: partial response.

* All patients were staged according to the American Joint Cancer Committee (AJCC) staging system 6th. # The thickness of esophageal wall at the lesion site.

Table S2. Clinical characteristics of 18 esophageal cancer patients with local recurrence after definite chemoradiotherapy with radiation simultaneous integrated boost.

No	Sex	Age	Tumor location	TNM stage *	With LR	Time (months)	Sites	Patterns
1	male	50	middle	T2N1M1b	Yes	12	SCV; subcarinal	In PTV66; In PTV54
2	male	53	upper	T3N1M0	No	14	TEG	Out of PTV54
3	male	64	upper	T3N1M1a	No	8	SCV	Out of PTV54
4	male	54	middle	T3N0M0	No	6	SCV	Out of PTV54
5	male	56	upper	T4N1M0	Yes	5	SCV	Out of PTV54
6	male	63	middle	T3N1M0	No	12	SCV; mediastinal	Out of PTV54; In-PTV54
7	male	56	middle	T3N0M0	No	10	TEG; mediastinal	Out of PTV54; In-PTV54
8	male	57	middle	T4N1M0	Yes	4	TEG	In PTV66
9	male	70	cervical	T2N1M0	No	10	SCV; mediastinal	Out of PTV54 In-PTV54
10	male	47	middle	T4N1M0	Yes	9	SCV	Out of PTV54
11	male	54	cervical	T4N0M0	No	3	SCV	Out of PTV54
12	male	64	upper	T3N1M1a	No	9	SCV	Out of PTV54

Abbreviations: LR: local recurrence; SCV: Supraclavicular; TEG: tracheoesophageal groove.

* All patients were staged according to the American Joint Cancer Committee (AJCC) staging system 6th.

Table S3. Clinical characteristics of 13 esophageal cancer patients with regional recurrence after definite chemoradiotherapy with radiation simultaneous integrated boost.

Parameters	Subgroups	No	%	5-y cumulative LRDP risk (%)	x^2	P
Age (years)	< 60	40	46	42	2.124	0.145
	≥ 60	47	54	26		
Sex	Female	20	23	11	4.957	0.026
	Male	67	77	41		
T stage	T1-2	17	20	30	0.251	0.616
	T3-4	70	80	34		
N stage	N0	31	36	24	1.955	0.162
	N1	56	64	39		
M stage	M0	74	85	33	0.090	0.765
	M1	13	15	36		
Clinical stage	II	29	33	25	1.688	0.194
	III-IV	58	67	38		
Middle thoracic	No	42	48	35	0.000	0.990
	Yes	45	52	32		
GTV-E (cm^3)	< 19	28	32	27	1.631	0.202
	≥ 19	59	68	36		
GTV-LN (cm^3)	< 3	65	75	31	1.783	0.182
	≥ 3	22	25	42		
LN number	< 2	55	63	34	0.000	0.988
	≥ 2	32	37	33		
Tumor length (cm)	< 4.7	36	41	25	3.416	0.065
	≥ 4.7	51	59	40		
Tumor thickness (cm)	< 1.62	41	47	32	0.317	0.573
	≥ 1.62	46	53	35		
Chemotherapy (cycles)	< 3	13	15	27	0.001	0.978
	≥ 3	74	85	34		
Response 20F	CR/PR	74	85	30	3.076	0.079
	SD	13	15	78		
Response 30F	CR	35	40	15	10.518	0.001
	PR/SD	52	60	48		

Abbreviations: GTV-E: the gross tumor volume of primary tumor at the esophagus; GTV-LN: the gross tumor volume of positive lymph nodes; CR: complete response; PR: partial response; SD: stable disease.

Survival comparison between subgroups of paitents of various parameters was performed by using the log-rank test. Parameters with P < 0.05 were highlighted in red.

Table S4. Univariate analysis for 5-year (5-y) cumulative risk of locoregional disease progression (LRDP) in esophageal cancer patients after concurrent chemoradiotherapy with simultaneous interated boost.

Variates	Subgroups	RR (95%CI)	P
Sex	Female	1	0.110
	Male	3.3 (0.8-14.3)	
Tumor length (cm)	< 4.7	1	0.092
	≥ 4.7	2.1 (0.9-5.0)	
Response-20F	CR/PR	1	0.421
	SD	1.5 (0.5-4.3)	
Response-30F	CR	1	0.023
	PR/SD	3.3 (1.2-9.2)	

Abbreviations: CI: confidence interval; GTV-E: the gross tumor volume of primary tumor at the esophagus; GTV-LN: the gross tumor volume of positive lymph nodes; CR: complete response; PR: partial response; SD: stable disease.

Reference groups were indciated with relative risk (RR) = 1. Parameters with P < 0.05 were highlighted in red.

Table S5. Multivariable cox regression analysis for 5-year (5-y) cumulative risk of locoregional disease progression in esophageal cancer patients after concurrent chemoradiotherapy with simultaneous interated boost.

Parameters	Subgroups	No	%	5-y cumulative DM risk (%)	x^2	P
Age (years)	< 60	40	46	31	1.004	0.316
	≥ 60	47	54	22		
Sex	Female	20	23	11	2.684	0.101
	Male	67	77	31		
T stage	T1-2	17	20	18	0.634	0.426
	T3-4	70	80	28		
N stage	N0	31	36	17	1.799	0.180
	N1	56	64	32		
M stage	M0	74	85	26	0.003	0.958
	M1	13	15	27		
Clinical stage	II	29	33	14	3.081	0.079
	III-IV	58	67	34		
Middle thoracic	No	42	48	28	0.003	0.954
	Yes	45	52	25		
GTV-E (cm^3)	< 19	28	32	19	1.288	0.256
	≥ 19	59	68	30		
GTV-LN (cm^3)	< 3	65	75	22	1.973	0.160
	≥ 3	22	25	38		
LN number	< 2	55	63	21	2.233	0.135
	≥ 2	32	37	35		
Tumor length (cm)	< 4.7	36	41	18	2.266	0.132
	≥ 4.7	51	59	33		
Tumor thickness (cm)	< 1.62	41	47	24	0.142	0.706
	≥ 1.62	46	53	28		
Chemotherapy (cycles)	< 3	13	15	24	0.077	0.781
	≥ 3	74	85	26		
Response 20F	CR/PR	74	85	25	0.334	0.563
	SD	13	15	47		
Response 30F	CR	35	40	9	9.952	0.003
	PR/SD	52	60	39		

Abbreviations: GTV-E: the gross tumor volume of primary tumor at the esophagus; GTV-LN: the gross tumor volume of positive lymph nodes; CR: complete response; PR: partial response; SD: stable disease.

Survival comparison between subgroups of paitents of various parameters was performed by using the log-rank test. Parameters with P < 0.05 were highlighted in red.

Table S6. Univariate analysis for 5-year (5-y) cumulative risk of distant metastasis (DM) in esophageal cancer patients after concurrent chemoradiotherapy with simultaneous interated boost.

Variates	Subgroups	RR (95%CI)	P
Sex	Female	1	0.271
	Male	2.3 (0.5-10.0)	
Clinical Stage	II	1	0.166
	III-IV	2.2 (0.7-6.6)	
Response-30F	CR	1	0.017
	PR/SD	4.5 (1.3-15.7)	

Abbreviations: CI: confidence interval; CR: complete response; PR: partial response; SD: stable disease.

Reference groups were indciated with relative risk (RR) = 1. Parameters with $P < 0.05$ were highlighted in red.

Table S7. Multivariable cox regression analysis for 5-year (5-y) cumulative risk of distant metastasis in esophageal cancer patients after concurrent chemoradiotherapy with simultaneous interated boost.

Parameters	Subgroups	No	%	5-y cumulative DP risk (%)	x^2	P
Age (years)	< 60	40	46	57	2.099	0.147
	≥ 60	47	54	41		
Sex	Female	20	23	21	6.734	0.009
	Male	67	77	57		
T stage	T1-2	17	20	41	0.822	0.365
	T3-4	70	80	51		
N stage	N0	31	36	34	4.139	0.042
	N1	56	64	57		
M stage	M0	74	85	47	0.659	0.417
	M1	13	15	58		
Clinical stage	II	29	33	32	5.483	0.019
	III-IV	58	67	58		
Middle thoracic	No	42	48	52	0.006	0.937
	Yes	45	52	47		
GTV-E (cm^3)	< 19	28	32	37	3.645	0.056
	≥ 19	59	68	55		
GTV-LN (cm^3)	< 3	65	75	43	5.860	0.015
	≥ 3	22	25	66		
LN number	< 2	55	63	45	1.026	0.311
	≥ 2	32	37	55		
Tumor length (cm)	< 4.7	36	41	38	4.383	0.036
	≥ 4.7	51	59	56		
Tumor thickness (cm)	< 1.62	41	47	46	0.576	0.448
	≥ 1.62	46	53	52		
Chemotherapy (cycles)	< 3	13	15	47	0.031	0.859
	≥ 3	74	85	49		
Response 20F	CR/PR	74	85	45	2.789	0.095
	SD	13	15	83		
Response 30F	CR	35	40	24	16.899	0.000
	PR/SD	52	60	66		

Abbreviations: GTV-E: the gross tumor volume of primary tumor at the esophagus; GTV-LN: the gross tumor volume of positive lymph nodes; CR: complete response; PR: partial response; SD: stable disease.

Survival comparison between subgroups of paitents of various parameters was performed by using the log-rank test. Parameters with P < 0.05 were highlighted in red.

Table S8. Univariate analysis for 5-year (5-y) cumulative risk of disease progression (DP) in esophageal cancer patients after concurrent chemoradiotherapy with simultaneous interated boost.

Variates	Subgroups	RR (95%CI)	P
Sex	Female	1	0.027
	Male	3.4 (1.2-9.8)	
N stage	N0	1	0.923
	N1	1.0 (0.4-2.4)	
Clinical Stage	II	1	0.354
	III-IV	1.6 (0.6-4.0)	
GTV-LN (cm^3)	< 3	1	0.062
	≥ 3	2.0 (1.0-4.3)	
Tumor length (cm)	< 4.7	1	0.367
	≥ 4.7	1.4 (0.7-2.9)	
Response-30F	CR	1	0.001
	PR/SD	3.7 (1.7-8.3)	

Abbreviations: CI: confidence interval; GTV-LN: the gross tumor volume of positive lymph nodes; CR: complete response; PR: partial response; SD: stable disease. Reference groups were indciated with relative risk (RR) = 1. Parameters with P < 0.05 were highlighted in red.

Table S9. Multivariable cox regression analysis for 5-year (5-y) cumulative risk of disease progression in esophageal cancer patients after concurrent chemoradiotherapy with simultaneous interated boost.

Parameters	Subgroups	No	%	5-y cumulative death risk (%)	x^2	P
Age (years)	< 60	40	46	50	1.005	0.316
	≥ 60	47	54	44		
Sex	Female	20	23	21	5.607	0.018
	Male	67	77	54		
T stage	T1-2	17	20	29	2.608	0.106
	T3-4	70	80	51		
N stage	N0	31	36	39	1.748	0.186
	N1	56	64	50		
M stage	M0	74	85	45	0.391	0.532
	M1	13	15	54		
Clinical stage	II	29	33	32	4.755	0.029
	III-IV	58	67	54		
Middle thoracic	No	42	48	44	0.523	0.470
	Yes	45	52	49		
GTV-E (cm^3)	< 19	28	32	25	7.186	0.007
	≥ 19	59	68	56		
GTV-LN (cm^3)	< 3	65	75	41	5.227	0.022
	≥ 3	22	25	64		
LN number	< 2	55	63	41	2.540	0.111
	≥ 2	32	37	56		
Tumor length (cm)	< 4.7	36	41	31	7.438	0.006
	≥ 4.7	51	59	57		
Tumor thickness (cm)	< 1.62	41	47	35	3.903	0.048
	≥ 1.62	46	53	56		
Chemotherapy (cycles)	< 3	13	15	54	2.116	0.146
	≥ 3	74	85	45		
Response 20F	CR/PR	74	85	41	14.629	0.000
	SD	13	15	79		
Response 30F	CR	35	40	20	16.895	0.000
	PR/SD	52	60	64		

Abbreviations: GTV-E: the gross tumor volume of primary tumor at the esophagus; GTV-LN: the gross tumor volume of positive lymph nodes; CR: complete response; PR: partial response; SD: stabled disease.

Survival comparison between subgroups of paitents of various parameters was performed by using the log-rank test. Parameters with P < 0.05 were highlighted in red.

Table S10. Univariate analysis for 5-year (5-y) cumulative risk of death in esophageal cancer patients after concurrent chemoradiotherapy with simultaneous interated boost.

Variates	Subgroups	RR (95%CI)	P
Gener	Female	1	0.149
	Male	2.2 (0.7-6.7)	
Clinical stage	II	1	0.813
	III-IV	1.1 (0.5-2.7)	
GTV-E (cm^3)	< 19	1	0.679
	≥ 19	1.3 (0.4-4.2)	
GTV-LN (cm^3)	< 3	1	0.160
	≥ 3	1.7 (0.8-3.5)	
Tumor length (cm)	< 4.7	1	0.111
	≥ 4.7	2.2 (0.8-5.5)	
Tumor thickness (cm)	< 1.62	1	0.765
	≥ 1.62	1.1 (0.5-2.5)	
Reponse-20F	CR/PR	1	0.007
	SD	3.1 (1.4-7.0)	
Reponse-30F	CR	1	0.020
	PR/SD	2.9 (1.2-7.1)	

Abbreviations: CI: confidence interval; GTV-E: the gross tumor volume of primary tumor at the esophagus; GTV-LN: the gross tumor volume of positive lymph nodes; CR: complete response; PR: partial response; SD: stable disease.

Reference groups were indciated with relative risk (RR) = 1. Parameters with P < 0.05 were highlighted in red.

Table S11. Multivariable cox regression analysis for 5-year (5-y) cumulative risk of death in esophageal cancer patients after concurrent chemoradiotherapy with simultaneous interated boost.
